# Becoming a Mentor: The Impact of Training and the Experience of Mentoring University Students on the Autism Spectrum

**DOI:** 10.1371/journal.pone.0153204

**Published:** 2016-04-12

**Authors:** Josette Hamilton, Gillian Stevens, Sonya Girdler

**Affiliations:** 1 School of Psychology and Speech Pathology, Curtin University, Perth, Western Australia, Australia; 2 Cooperative Research Centre for Living with Autism Spectrum Disorders (Autism CRC), Long Pocket, Brisbane, Queensland, Australia; 3 School of Occupational Therapy and Social Work, Curtin University, Perth, Western Australia, Australia; Nagoya University Graduate School of Medicine, JAPAN

## Abstract

While it is widely recognised that the number of young adults diagnosed with Autism Spectrum Disoders (ASD) is increasing, there is currently limited understanding of effective support for the transition to adulthood. One approach gaining increasing attention in the university sector is specialised peer mentoring. The aim of this inductive study was to understand the impact of peer mentor training on seven student mentors working with university students with an ASD. Kirkpatrick’s model framed a mixed methods evaluation of the mentors’ training and description of their experience. Overall, the training was well received by the mentors, who reported on average a 29% increase in their ASD knowledge following the training. Results from the semi-structured interviews conducted three months after the training, found that mentors felt that the general ASD knowledge they gained as part of their training had been essential to their role. The mentors described how their overall experience had been positive and reported that the training and support provided to them was pivotal to their ability to succeed in as peer mentors to students with ASD. This study provides feedback in support of specialist peer-mentoring programs for university students and can inform recommendations for future programs and research.

## Introduction

Autism Spectrum Conditions (ASD) are a group of lifelong developmental conditions that impact on a person’s ability to communicate, understand and interact with the social and physical world [1]. ASD is estimated to impact on the lives of 1% of the school-aged [2] and adult populations [3]. While intellectual disability commonly co-occurs with ASD it is likely that 60% of people living with ASD have normal or above average intelligence [4]. This group is commonly referred to as having High Functioning Autism (HFA). Despite the label HFA, these individuals still experience many challenges and are at risk of poor outcomes in adulthood in many life areas, including employment, social relationships, health status and overall quality of life [5]. While their challenges are well documented, there is a dearth of evidence exploring the most effective available supports [5].

Like their peers, many young adults with HFA aspire to attend university. University environments are likely to be particularly appealing for those with HFA given their propensity for specialised interests and aptitude for academic pursuits. However, the university environment presents many challenges to young adults with HFA [6], evident in findings from a recent national Australian survey of 313 adults with HFA reporting that only 13% held a university degree, a proportion substantially lower than the 2011 national average of 25% [1]. The majority of respondents to this survey identified that although their need for support in higher education was significant, their needs were unmet in the areas of learning support (78%), behaviour support (83%) and social support (84%) [1]. Nearly half (42%) of the respondents to this survey acknowledged that negative social experiences such as being teased, bullied or socially excluded were the worst aspects of their educational experience.

Collectively, these findings suggest that there is a need for more specialised services to meet the individual and complex support needs of students with ASD [1] ultimately enhancing the experience and promoting the retention of university students with HFA. In part, because of its social nature, peer mentoring is one approach which may be effective in meeting the support needs of students with ASD [6–8]. As noted by Crisp and Cruz [9] in their critical review of literature on mentoring college students mentoring is not a new concept, but lacks a widely accepted definition and theoretical understanding of how mentoring relationships work. Roberts [10] defined mentoring as a “formalized process whereby a more knowledgeable and experienced person actuates a supportive role of overseeing and encouraging reflection and learning within a less experienced and knowledgeable person, so as to facilitate that persons’ career and personal development” (p.162). Jacobi [11] outlined that researchers tended to agree that mentoring relationships were personal and reciprocal in nature, and centred on the growth and accomplishment of the mentee through broad forms of support. While research is limited, peer mentoring programs have been associated with improvements in academic outcomes and self-regulation in college students with Asperger’s syndrome [12]. Research has also emphasised that the mentee’s experience is impacted by factors including their mentor’s approach and self-efficacy [13], the mentor-mentee relationship [14] and the quality and nature of mentor supervision [15, 16]. While incomplete, research to date has highlighted the importance of adequate training and support of mentors in ensuring effective interpersonal relationships between mentors and mentees with an ASD [16]. It is likely that these supports are critical to the success of such programs given the interpersonal challenges experienced by young adults with ASD [17].

Despite the acknowledged importance of training and ongoing support for mentors in peer mentoring programs [18], there is a paucity of research examining the education of mentors, and none that specifically evaluates the training and support needs of peer mentors for students with an ASD. The current research provides a formative, responsive evaluation of the training received by student mentors working with university students diagnosed with a HFA at Curtin University, Western Australia, during first semester 2014. It explores the experiences of student mentors and identifies the elements of training and support important in the provision of an efficacious mentoring program.

## Method

### Study design

Kirkpatrick’s [19, 20] four level model [21] of training evaluation which examines participant satisfaction, participant learning, application of knowledge into practice, and outcomes achieved provided a framework for this study. As the four levels are not ordered hierarchically this model provides a flexible structure that can be tailored according to the needs of a specific evaluation. In particular, the third level: application of knowledge in practice, has been highlighted as critical in evaluating and refining training programs for specific contexts [20]. The current evaluation employed three levels of this model (Table 1), which was operationalised using a mixed methods approach [22], incorporating both descriptive quantitative and qualitative data, obtained through semi-structured interviews. The impact of the mentoring program on students with HFA is the topic of another publication.

**Table 1:** Data Collection.

Level of Evaluation	Number of Participants	Time Administered	Evaluation Method	Outcome Measured
1	5	Immediately post-training	Questionnaire	Participant satisfaction with training.
2	5	Immediately pre and post training	Questionnaire	Participant learning measured by change in knowledge of ASD.
3	7	3 months post- training	Semi-structured interview	Application of knowledge to practice

### Training and peer mentoring intervention

In response to the increasing numbers of students with HFA and concern that the complex and individualised needs of these students were not being meet by generic disability supports, in the first semester (24^th^ February–27^th^ June) of the 2014 academic year Curtin University in Western Australia implemented the Curtin Specialist Peer Mentoring Program (CSPM). The aim of the CSMP was to increase the retention rate, academic performance, social success and wellbeing in university education settings for students with HFA.

Psychology and Occupational Therapy Masters and undergraduate students were canvassed through presentations in lectures as to their interest in being a mentor to a student with HFA. Interested students were asked to submit their resume’s to the two CMSP program coordinators (an education specialist and psychologist both experienced in working with young adults with HFA) and assessed for the suitability. In preparation for their role, student mentors attended a four hour course of generic mentor training (delivered by university disability staff). In addition, CSMP mentors attended six hours of training specifically aimed at understanding and mentoring students with HFA. This training was delivered by the CSMP program coordinators. The delivery of the training included a power point presentation, question and answer sessions, and a panel discussion involving the CSMP coordinators and a student with HFA who had participated in a generic mentor program and graduated from university. During the last 30 minutes of the training day ‘matched’ mentors and mentees were introduced. Mentees were encouraged to bring a support person to this session. Mentors and mentees were matched by the program coordinators based similarity in backgrounds and interests (including sports or special interests), with some mentees expressing a preference for a male or female mentor. All students attending Curtin University who had declared their diagnosis of ASD to Disability Services were eligible to be mentees in the CSMP.

During the semester, mentors met one-on-one with their mentees for up to two hours per week and attended a weekly one hour group supervision meeting, facilitated by the two CSMP coordinators. In addition, two student mentors facilitated an on-campus weekly social-group meeting for students with HFA and their mentors where mentees and their mentors interacted to learn and practice social skills as a group. Throughout the year all CSMP mentors received remuneration for their time at an hourly rate of $30.00(AUD) per hour, usually for between 3 to 4 hours per week during semester and a certificate at the end of the year to acknowledging their involvement in the program.

### Participants

All student mentors who attended the generic training and specific ASD mentor training were eligible to participate in this study. Of the 10 students who were eligible seven consented to participate in this study (see Table 1). Five participants were Masters students (two from the School of Psychology and three from the School of Occupational Therapy) and two were undergraduate students from the School of Psychology. The CSMP actively recruited students from these areas as they were considered the most likely to have the appropriate skills to mentor students with HFA. Of the seven participants, four had previous experience in working with people with ASD and two had previous experience in mentoring students with physical disabilities. Six participants were female. The age of participants ranged between 21 and 30 years, with a mean age of 26 years. The mentors’ time of enrolment at university varied between one year and eight years, with a mean of 2.9 years.

Five participants mentored the students with HFA on an individual basis, while two participants facilitated the weekly social groups. The two undergraduate students who facilitated the weekly social groups were recruited during level three of the evaluation, i.e. at the interview stage, and did not complete levels one or two of the evaluation. This was deemed appropriate as while these mentors attended the generic and specific mentor training and mentored students with HFA at the group level in their roles as facilitators of the social group, they did not have experience with individual mentoring, and were therefore unable to report on the relevance of their training to the mentoring role at the individual level, as evaluated by levels 1 and 2, but on how their training had impacted on their experience as a student mentors at the group level, as evaluated in level 3. All participants attended the weekly group supervision meetings.

### Measures

#### Level 1 –Participant satisfaction

Participant (n = 5) satisfaction was measured via a purpose designed satisfaction questionnaire administered immediately following the training session. This questionnaire included seven Likert scaled questions (presented in Table 2) and three short answers requesting comment on aspects of the training that were the most and least valuable, and feedback of what could be improved in future training. All responses were anonymous to minimise the potential of bias.

**Table 2:** Mean Response to Participant Satisfaction Questions.

Questions	Average Score
1. Course ‘Learning Outcomes’ clearly stated.	4.6
2. Content of the course was directly relevant to the learning outcomes.	4.8
3. The instructor was an effective communicator.	4.8
4. The instructor was well prepared.	5
5. The course materials were clearly written, well presented and easy to use.	4.6
6. I intend to apply much of the material to my job.	4.8
7. The venue was appropriate and provided a good learning environment.	4.4
**Overall Mean**	4.7

#### Level 2 –Participant learning

A pre-test post-test training questionnaire measured mentor (n = 5) learning from the training experience. This comprised three short scenarios common to students with ASD (including orientating themselves to the university campus and its facilities, assignment deadlines and class attendance, making friends at university), and asked participants to identify the likely difficulties experienced by students with ASD and suggest strategies to assist.

#### Level 3 –Application of knowledge

To evaluate the application of knowledge, participants (n = 7) took part in a semi-structured interview comprising of six open-ended questions (see Appendix A), approximately three months after the training. Participants were encouraged to reflect upon their initial training and its effectiveness in preparing them for their role as a student mentor, as well as any support or difficulty that they had in applying their knowledge within their role. Interviews also explored the experience of the participants in mentoring students with HFA.

### Data analysis

#### Level 1 –Participant satisfaction

The average score for each of the seven scaled questions was calculated as an overall average score and the information gained from the three short answer questions was analysed using content analysis [23].

#### Level 2 –Participant learning

Answers were scored against a marking key with a maximum of 10 points for identifying difficulties and 10 points for identifying strategies (20 points maximum for each scenario) across three scenarios, for a total score of 60. Average pre-test post-test scores were compared, in order to determine whether the training had led to an increase in knowledge of working with individuals with an ASD.

#### Level 3 –Application of knowledge

Semi-structured interviews were digitally recorded and transcribed verbatim. The transcribed interviews were de-identified and imported into the NVivo software which assisted with data management. Open coding as described by Strauss and Corbin [24] guided the naming, comparison and categorising of data. Coded data was then grouped into broad categories and scrutinised in relation to similarities and differences [24]. These broad categories were then systematized into themes [24, 25].

Trustworthiness of the findings was established via multiple strategies. In order to ensure credibility of the findings, the researcher summarised and paraphrased the interview content at the completion of each interview, giving interviewees the opportunity to validate or correct interpretations [26]. The credibility of the analysis process was further enhanced by crosschecking of the analysis process by an experienced qualitative researcher. A record of the conceptual development of the study from data collection, to coding decisions, data analysis processes and critical decision making was also maintained throughout the research [27]. Reflective journaling allowed for the uncovering of personal biases and provided a record of reflections and key decision making in relation to the analysis [27].

### Ethical considerations

The study was approved by the Curtin University Human Research Ethics committee in Western Australia (HREC approval number PSYCH SP 2014‐04). Written consent to participate was obtained from all participants. Study procedures and confidentiality of records were maintained in line with the Declaration of Helsinki.

## Results

### Level 1: Participant satisfaction

Results (n = 5) from the participant satisfaction questionnaire indicated that the training was well received. Average responses to each of the first 7 questions ranged from 4.4 to 5 out of 5, with 5 being the most favourable response. Overall, the mean satisfaction score from these questions was 4.7. There were no outliers. Table 2 provides the average score for each item.

Although satisfaction scores indicated that the experience of attending the training day was positive for participants, the three short answer questions elicited further information related to training satisfaction. Participants highlighted that the interpersonal aspects of the training were the most beneficial; specifically the extensive experience of the program coordinators in working with individuals with HFA and their sharing of experiences ‘*that were not on the slides’*. Participants also valued the *‘interactions between mentors*, *and the exchange of ideas’*. Participants suggested that the training could be improved by allowing more time, the use of video scenarios to enhance participants’ understanding of individuals with HFA and a greater emphasis on how to practically apply their learning to their mentoring role.

### Level 2: Acquisition of knowledge

The average score on the pre-training questionnaire was 20 out of a possible 60 and the average post-training score was 25.8, indicating an average 29% increase in scores. Although there appeared to be a large discrepancy in participant learning (as the range of percentage change from pre-to post-test scores varied from 0% to 100%), this was reflective of the participants entering into the training who had varying levels of knowledge and experience in working with individuals with an ASD, form participants who had no experience to participants with significant experience. Three participants scored less than 5% change, one had a 74% increase and one a 100% increase in scores. Despite this, all participants rated the training as a valuable experience, indicating that acquisition of knowledge is not the only factor of importance when considering training effectiveness.

### Level 3: Application of Knowledge

Semi-structured interviews were conducted approximately three months after the training was completed. These provided insight into the complexities of working as a student mentor for students with HFA. At this stage, participants still found it difficult to articulate the specific aspects of training that they were able to apply to their mentoring role, with most stating *‘all of it’* was useful; however, some noted that certain elements were not specifically applicable to their mentee. Many participants reflected that the highlight of the training was the talk given by a guest speaker with HFA who had completed her undergraduate degree. The personal insight shared by this speaker in relation to her experiences of being a university student with HFA was considered invaluable when learning about their role as peer mentors. Participants suggested that future iterations of the training should include more examples of first-person insights.

All participants highlighted specific areas of the training as being essential to their role as a student mentor. These included general ASD knowledge, the weekly supervision meetings, the mentor-coordinator relationship, overall communication between mentor and the coordinators, role definition and the provision of resources. Knowledge of ASD was described as particularly important.

#### ASD knowledge

Many mentors reported that the training, which was provided before they started working, increased their knowledge about and insight into what life may be like for a person with ASD. The knowledge and experience of the coordinators and the personal experience shared by a student with HFA during the training were highlighted by mentors as the most influential sources of knowledge. Knowledge regarding particular aspects of the experience of having an ASD were emphasised by mentors as having a great impact on their ability to embark on their role. Specifically, this knowledge included: the sensory differences inherent in ASD, the experience and prevalence of anxiety and the underlying cognitive processes that may be experienced. These areas are described below.

#### Sensory differences

Most mentors stated that they were previously unaware of the sensory differences experienced by people with ASD and that the information they learnt as part of their training helped them to understand the experience of their mentees. This knowledge also helped them to support their mentees more effectively:

*Everyone was making a lot of noise*, *and [my mentee] really didn’t enjoy that*, *but it was good to know … So that was a good example of … applying that knowledge in the situation*, *“Oh*, *so that’s why this is bad*. *That’s why they’re kind of freaking out”*.

Participants considered that information about the sensory challenges of people with ASD was imperative and should be included in future iterations of the program. Participants also reported that they valued the discussion on what they might expect regarding sensory differences, as well as what it might look like if these differences were impacting on an individual and the specific strategies that may be helpful in assisting their mentees.

#### Anxiety

Many mentors expressed surprise at the prevalence of anxiety among individuals with an ASD. They reported that the training alerted them to the fact that, although their mentee may not appear anxious, it was likely that they were, e.g. ‘*I didn’t realise that social anxiety was such a common experience for people on the autism spectrum*.*’*

Some mentors reported that their default position now, was to assume that their mentee was impacted by anxiety and modified their approach accordingly. This anticipatory and empathic approach to mentoring students with ASD has been reported, previously as being the most likely to be effective [13], particularly given the heightened anxiety that those with ASD are likely to experience during the transition into higher education [6].

#### Underlying cognitive processes

The mentors described that understanding the cognitive processes that underlie the seemingly simple everyday tasks difficulties with initiation and a need for routine, helped them to empathise with their mentee, as well as establish an effective mentor-mentee relationship and provide individualised support:

*For example it takes a long time to initiate something … that they get very fatigued because all the time they are thinking*, *thinking*, *thinking*, *thinking … I just thought you know*, *might be difficulty with a*, *b and c*.

The mentors highlighted the importance of their training in helping them to understand the social experiences of an individual with ASD, which impact on their understanding and navigation of the social world. Several mentors mentioned that they had not thought about the impact that a lack of experience in certain common situations, such as ordering food or drink in a café, may have on their mentees ability to cope with university life;

*At Uni*, *a lot of us have had experiences at school*, *or*, *other circumstances where … that worked in that situation*, *so I’ll use that*. *Whilst [my mentee] doesn’t have that at all so a lot of things*, *new experiences*, *they’re brand new*, *[my mentee has] got no way of knowing how to deal with it*.

### Factors Impacting on the Experience of Mentors

Across the semi-structured interviews the mentors commonly described weekly meetings, mentee characteristics, the mentor-coordinator relationship, communication, role definition and resources as factors that impacted on their experience as mentors.

#### Weekly meetings

For the mentors the weekly group supervision meetings with the program coordinators were described as vital in enabling them to provide high quality mentorship to their mentees. For example, they were described as being able to ‘*give you encouragement*, *to hear what other people are doing*, *what they’re going through*,*’* and *‘I think you wouldn’t be able to get away with not having them*.*’*

Weekly supervision meetings gave mentors the chance to debrief, brainstorm, share resources, receive support, alleviate isolation, and reflect on their work and the progress of their mentee(s). ‘*I think [weekly supervision is] a really important part because that’s where I get most of my ideas of how to work out different situations that arise*.*’*

Weekly supervision was also considered important in helping the mentors to balance their aspirations and apprehensions for their mentee: *‘If there wasn’t someone to say*, *you know you are doing the right thing*, *I probably would have felt a bit of a fraud and felt that I was of no benefit to [mentee] whatsoever*.*’*

#### Mentee characteristics

Throughout the interviews, the participants perceived that the mentees who had increased self-awareness and were more motivated to engage socially, were easier to work with. Mentors with more sociable mentees described their role in more positive terms.

*I found one of the big differences was our relationships with our mentees … a lot of them are a lot more self-aware of what [ASD] is … I suppose they were a lot more receptive to getting help with different things*. *Whilst if you have someone who perhaps isn’t as aware … they don’t think they need any assistance with anything*, *which makes it harder*.

In contrast, mentees who were less sociable, had less insight into their difficulties, or who were less motivated to change, were perceived by mentors as being more challenging to work with. Mentors working with these students reported that the face-to-face support, provided in the group supervision meetings was paramount in supporting their role, e.g. *‘you get a lot of support from [the coordinator]*, *saying stick with it*, *you know*, *even though you’re not getting a response from the mentee*.*’*

#### Mentor-coordinator relationship

Participants reported that developing a strong relationship with the program coordinators was vital to succeeding in their role as a mentor. This relationship was strengthened by the shared communication and respect between mentors and coordinators and the experience and knowledge of the coordinators in working with people with ASD. Specifically, this relationship was facilitated by the collaborative nature of the group meetings, the respect shown to mentors in developing their role, the coordinators’ quick responses to any concerns raised and the caring attitude that coordinators displayed toward both mentors and mentees.

*The way that they’ve run the weekly meetings … so collaboratively*, *and every bit of information they gave us was useful and applicable*.

*I was able to call [coordinator] and immediately she … acted upon the situation*.

*You can tell they care*, *which I think is important as well*, *… they do care about how we’re going and how the mentees are going*.

#### Communication

The importance of good communication was a key theme that emerged from the interviews. As a part of their role, mentors completed a weekly report which they submitted prior to the weekly meeting. Participants commented that the coordinators *‘obviously read them’* and discussed the issues raised in the weekly team meeting. This approach enhanced the mentors’ self-confidence and gave them a sense that their role was valued by the University.

The immediacy of the coordinators’ response to participants’ email or telephone enquiries was essential in enabling the mentors to respond in a competent and timely manner to their mentees needs. This level of support also alleviated the mentors isolation, as although the largely worked independently, they described feeling valued and *‘part of a team*.*’* Mentors reported that initially there had been some communication difficulties during the early stages of the program, *‘that was probably the biggest barrier*, *the communication between all of us at the beginning*.*’* Difficulties with communication were seen as a barrier to feeling part of a team. However, these communication difficulties *‘resolved over time’* as the peer mentoring team established effective working patterns.

#### Role definition

The mentors in this study acknowledged that initially they had experienced some anxiety in their new role as a peer mentor to a student with HFA: *‘At the start you didn’t really know what it was that you were meant to be doing*, *because we didn’t really know what the mentee needed you for*.*’*

Several mentors expressed their concerns in relation to defining the boundaries and scope of their role: ‘*I wasn’t sure if it was my role*, *or my job*.*’* The mentors described the social group, as vital to the success of the program, and as their peer mentoring and the social group serving *‘two very separate and good purposes*.*’* The role of the coordinators’ was also highly valued. Mentors described the importance of having trusted coordinators able to support their roles, as well as the mentees. Their role was described as advocating for, and providing, resources, as well as navigating organisational policies: ‘*the resources they bring in have been very helpful … [The coordinators] have a bit more power than me and was able to speak to the right person*.*’*

#### Resources

The mentors highlighted the importance of the resources they received such as work sheets, brochures and service information in supporting their mentoring role. These resources were important in shaping the mentors work with mentees, helping them to be more efficient and increase their understanding of the challenges their mentees were experiencing.

*Every week [during supervision] there are new things … that we can actually do hands on with our mentees’ … the resources [the coordinators] give use have been very helpful*.

#### The overall mentor experience

The mentors in this study reported that their overall experience of mentoring peers with an HFA was overwhelmingly positive. The support provided to them through the CSMP was integral to this experience. Many mentors commented on the professional and personal learning they had gained from their experience as student mentors: ‘*I feel like I’ve got probably more out of it than [my mentee] has*.*’*

## Discussion

This study explored the impact of peer mentoring training on the mentoring roles of university students working with mentees with HFA. Consistent with findings from previous research the mentors in this study highlighted the importance of ASD specific training in supporting their role [17]. One target of the training was providing information on the sensory processing difficulties commonly experienced by people with ASD [28] which helped the mentors to better understand and support their mentee in a university environment. Understanding the cognitive processes common in people with ASD was also central in mentors developing effective working relationships with their mentees. Understanding how people with ASD perceive everyday life has previously been shown to be important in developing the anticipatory, empathic and logical approach purported to be most effective when working with this group [29]. Collectively, these findings suggest that increasing mentors’ knowledge of the specific and often highly individualised needs of people with HFA should be prioritised in future specialised peer mentoring programs.

The mentors also pointed to features of the peer mentoring program that enhanced their experiences. Consistent with findings from previous mentoring program evaluation the group approach to supervision facilitated by experienced program coordinators was described as a main factor in enabling successful mentor-mentee relationships [16]. As with other mentors, participants in this study valued the regular contact with the program coordinators, and the frequent exchanges of information, ideas and experiences with the other mentors and coordinators [17, 30, 31]. These weekly sessions were also key in helping the mentors to balance their aspirations and apprehensions for their mentee [16].

While the interpersonal aspects of working with a person with ASD can be challenging [17], supports such as weekly supervision can provide a mechanism for feedback and result in more effective mentor-mentee relationships [16, 30, 31]. Frequent communication between mentors and coordinators is key to supporting both mentors [16] and people with ASD [17]. Establishing a new role as a mentor can cause anxiety [16] and this study found that the strong relationship between the coordinators and mentors was central in managing this anxiety. While the mentors described their experience as positive, it is likely that this was at least in part the result of the training and support available to them in their roles. Planning for peer mentoring programs for students with HFA must not only consider the needs of mentees, but also the support and training needs of mentors. In particular explicitly addressing communication in mentor training, providing mentors with a written protocol for dealing with conflict and discussing and practicing conflict resolution strategies may provide some support to mentors in addressing communication barriers.

The mentor-mentee relationships described in this study were influenced by both the personal attributes of the mentors and mentees. Consistent with previous research, mentee characteristics impacted on the self-efficacy of mentors [14], findings which highlight the reciprocal nature of this relationship. This points to the importance of considering how mentors and mentees with HFA are matched in future programs, with consideration of factors such as personality fit and the potential for areas of common interest. Clearly, as highlighted in this study, the mentor-mentee relationship is also influenced by environmental factors such as supervision, perceived organisational support and available resources [17, 32, 33], suggesting the structural supports are also critical to the success of such programs. These findings provide a directive for the planning of future programs, in that the role of supports and resources in determining the ultimate success of such programs must not be underestimated.

While this study was limited in its sample size, the use of Kirkpatrick’s [19–21] model of training evaluation which framed the mixed methods approach, allowed for both an in-depth understanding of the mentors’ satisfaction, and how they applied their new knowledge to their mentoring role [34]. Findings from the semi-structured interviews, which examined the experiences of mentors and aimed to understand those factors that contributed to the success of the CSMP from the mentors’ perspective, were particularly rich. This study did not explore if the student mentors used sources of support outside of the CSMP to help them cope or understand their mentoring experience. Clearly, future research is needed to understand those factors that contribute to successful outcomes for both mentors and mentees. Potential outcomes for measurement include for mentors could include changes in self-efficacy in relation to mentoring, the impact of being a mentor on personal development and the learning gained from the mentoring experience. For mentees with HFA potential outcomes of interested could be anxiety, communication apprehension and competence, and information regarding academic success and retention.

In a service environment where evidence-based interventions which support the needs of university students with HFA are profoundly needed [8], the findings from this study will make an important contribution to informing future specialised peer mentoring programs. It is hoped that these findings will encourage more universities to be not only proactive in enrolling and supporting students with HFA, but also recognise the potential benefits for students both with and without HFA from participating in peer mentoring programs, such as the one described in this study.

## Conclusion

While peer mentoring has been proposed as one approach particularly suited to meeting the complex and individualised needs of this group, the development of these programs has to date been hindered by a lack of research examining the training needs and experiences of student mentors. Successful peer mentoring programs for students with ASD are dependent on ensuring that needs of both mentors and mentees are met. Overall, findings from this study revealed that the experience of being a mentor to university students with HFA was an overwhelming positive experience for participants, suggesting that such programs have many benefits for both mentors and mentees.

## Appendix A: Level Three, Mentor Training Evaluation Interview Schedule

- Please describe any changes in your understanding of Autism Spectrum Disorders that you believe are directly due to the training program.
- What skills/knowledge from the training did you find most useful?
- What skills/knowledge from the training did you find least useful?
- How have you been able to apply the skills learnt in the training program to your work as a student mentor?
- Have you had difficulty applying any skills learned to your work? If so, why do you perceive this to be?
- How would you make the training better? What would you like to see included in the training?
- Is there anything else you would like to say about the training or your role as a student mentor?

## References

[1] Autism Spectrum Australia (Aspect). We Belong: Investigating the experiences, aspirations and needs of adults with Asperger's disorder and high functioning autism Sydney: Autism Spectrum Australia (Aspect); 2012.

[2] Baron-Cohen S, Scott FJ, Allison C, Williams J, Bolton P, Matthews FE. Prevalence of autism-spectrum conditions: UK school-based population studies. The British Journal of Psychiatry. 2009;194(6):500–9. 10.1192/bjp.bp.108.059345 19478287

[3] Brugha TS, McManus S, Bankart J, Scott F, Purdon S, Smith J. Epidemiology of autism spectrum disorders in adults in the community in England. Archives of General Psychiatry. 2011;68(5):459–66. 10.1001/archgenpsychiatry.2011.38 21536975

[4] Chakrabarti S, Fombonne E. Pervasive Developmental Disorders in Preschool Children: Confirmation of High Prevalence. American Journal of Psychiatry. 2005;162(6):1133–41. 15930062 10.1176/appi.ajp.162.6.1133

[5] Howlin P, Moss P, Howlin P, Moss P. Adults With Autism Spectrum Disorders. Canadian Journal of Psychiatry. 2012;57(5):275–83. 22546059 10.1177/070674371205700502

[6] Hastwell J, Harding J., Martin N., Baron-Cohen S. Asperger Syndrome Student Project, 2009–12: Final Project Report. Cambridge: University of Cambridge, 2013.

[7] Bebko JM, Schroeder JH, Ames ME. A Mentoring Program for Students with Asperger and ASDs. Toronto, Ontario: York University, 2011.

[8] Van Hess V, Moyson T, Roeyers H. Higher education experiences of students with Autism Spectrum Disorder: Challenges, benefits and support needs. Journal of Autism and Developmental Disorders. 2015;45:1673–88. 10.1007/s10803-014-2324-2 25448918

[9] Crisp G, Cruz I. Mentoring College Students: A Critical Review of the Literature Between 1990 and 2007. Research in Higher Education. 2009;50:525–45.

[10] Roberts A. Mentoring Revisited: a phenomneological reading of the literature. Mentoring and Tutoring. 2000;8(2):145–70.

[11] Jacobi M. Mentoring and Undergraduate Academic Success: A Literature Review. Review of Educational Research. 1991;61(4):505–32.

[12] Ness BM. Supporting Self-Regulated Learning for College Students with Asperger Syndrome: Exploring the “Strategies for College Learning” Model. Mentoring & Tutoring: Partnership in Learning. 2013;21(4):356–77. 10.1080/13611267.2013.855865

[13] Martin SM, Sifers SK. An evaluation of factors leading to mentor satisfaction with the mentoring relationship. Children and Youth Services Review. 2012;34(5):940–5. 10.1016/j.childyouth.2012.01.025.

[14] Young AM, Perrewé PL. The exchange relationship between mentors and protégés: The development of a framework. Human Resource Management Review. 2000;10(2):177–209.

[15] Larose S. Trajectories of Mentors’ Perceived Self-Efficacy during an Academic Mentoring Experience: What They Look Like and What are their Personal and Experimental Correlates? Mentoring & Tutoring: Partnership in Learning. 2013;21(2):150–74. 10.1080/13611267.2013.813728

[16] Rajuan M, Tuchin I, Zuckermann T. Mentoring the mentors: First-order descriptions of experience-in-context. The New Educator. 2011;7(2):172–90.

[17] Mavropoulou S, Avramidis E. Befrienders to persons in the autistic spectrum in Greece: what support do they offer and what challenges they face? European Journal of Special Needs Education. 2012;27(3):337–53. 10.1080/08856257.2012.691230

[18] O’Shea S, Harwood V, Kervin L, Humphry N. Connection, Challenge, and Change: The Narratives of University Students Mentoring Young Indigenous Australians. Mentoring & Tutoring: Partnership in Learning. 2013;21(4):392–411. 10.1080/13611267.2013.855863

[19] Kirkpatrick D. Great ideas revisited. Training and Development. 1996;50(1):54–9.

[20] Kirkpatrick J. The hidden power of Kirkpatrick's four levels. Training and Development. 2007;61(8):34.

[21] Winfrey EC. Kirkpatrick's Four Levels of Evaluation In: Hoffman B, editor. Encyclopedia of Educational Technology. San Diego (CA): College of Education; 1999.

[22] Cresswell JW, Plano Clark VL. Designing and Conducting Mixed Methods Research. Thousand Oaks (CA): Sage Publications, Inc; 2011.

[23] Hsieh H, Shannon SE. Three approaches to qualitative content analysis. Qualitative Health Research. 2005;15(9):1277–88. 16204405 10.1177/1049732305276687

[24] Strauss A, Corbin J. Basics of qualitative research: grounded theory procedures and techniques Newbury Park (CA): SAGE Publications Inc; 1990.

[25] Crotty M. Phenomenology and nursing research. South Melbourne (Australia): Churchill Livingston; 1996.

[26] Lincoln YS, Guba EG. Naturalistic Inquiry. Newbury Park, CA: SAGE; 1985.

[27] Grbich C. Qualitative Research in Health: An Introduction. St Leonards, New South Wales: Allen & Unwin Pty Ltd; 1999.

[28] Tavassoli T, Miller LJ, Schoen SA, Nielsen DM, Baron-Cohen S. Sensory over-responsivity in adults with autism spectrum conditions. Autism. 2013:1362–3613. 10.1177/1362361313477246 24085741

[29] Martin N, Beardon L, Hodge N, Goodley D, Madriaga M. Towards an inclusive environment for university students who have Asperger syndrome (AS). The Journal of Inclusive Practice in Further and Higher Education. 2008;1(1):3–14.

[30] Morton S. What support do health visitor mentors need? Community Practitioner. 2013;86(8):32–5. 23986990

[31] Parise MR, Forret ML. Formal mentoring programs: The relationship of program design and support to mentors’ perceptions of benefits and costs. Journal of Vocational Behavior. 2008;72(2):225–40. 10.1016/j.jvb.2007.10.011.

[32] Baranik LE, Roling EA, Eby LT. Why does mentoring work? The role of perceived organizational support. Journal of Vocational Behavior. 2010;76(3):366–73. 10.1016/j.jvb.2009.07.004. 20401322 PMC2855142

[33] Ensher EA, Murphy SE. The Mentoring Relationship Challenges Scale: The impact of mentoring stage, type, and gender. Journal of Vocational Behavior. 2011;79(1):253–66. 10.1016/j.jvb.2010.11.008.

[34] Bolte S. The power of words: Is qualitative research as important as quantitative research in the study of autism? Autism. 2014;18(2):67–8. 24596959 10.1177/1362361313517367

